# Extracorporeal Cardiac Shock Wave Therapy Ameliorates Clinical Symptoms and Improves Regional Myocardial Blood Flow in a Patient with Severe Coronary Artery Disease and Refractory Angina

**DOI:** 10.1155/2009/639594

**Published:** 2009-08-20

**Authors:** Christian Prinz, Oliver Lindner, Thomas Bitter, Detlef Hering, Wolfgang Burchert, Dieter Horstkotte, Lothar Faber

**Affiliations:** ^1^Department of Cardiology, Heart and Diabetes Center North Rhine-Westphalia, Ruhr University Bochum, 32545 Bad Oeynhausen, Germany; ^2^Institute of Radiology, Nuclear Medicine and Molecular Imaging, Heart and Diabetes Center North Rhine-Westphalia, Ruhr University Bochum, 32545 Bad Oeynhausen, Germany

## Abstract

Different therapeutic options are being used for chronic coronary artery disease (CAD). We report about a 51-year-old female with CAD and refractory angina pectoris despite maximally tolerated medical therapy and after both percutaneous coronary intervention (PCI) and coronary artery bypass grafting (CABG). The patient received cardiac shock wave therapy (CSWT) over a period of 6 month. There was no arrhythmia during or after treatment; enzyme levels were normal at all times. PET imaging showed a substantial improvement of myocardial stress perfusion. Since the patient reported that she now was fully capable to deal with her everyday life, further treatment options were postponed. Our case report suggests that ultrasound-guided CSWT is able to improve symptoms and perfusion in ischemic myocardium.

## 1. Introduction

Different therapeutic options are being used for chronic coronary artery disease (CAD), including medical treatment, percutaneous coronary intervention (PCI), and coronary artery bypass grafting (CABG). However, a growing number of patients exists who suffer from disabling angina despite, or after having undergone, all these modalities. For this challenging subset of patients with so-called chronic refractory angina, several alternative therapies such as neurostimulator implantation, laser revascularisation, or angiogenesis by gene or cell therapy [1] may be effective. However, these modalities are invasive, expensive, or still at a preclinical stage. Ultrasound-guided cardiac shock wave therapy (CSWT [2]), on which we report now, is another modality to deal with chronic refractory angina. We used CSWT to improve myocardial ischemia in a patient with severe CAD and refractory angina pectoris.

## 2. Case Presentation

A 51-year-old female with CAD, arterial hypertension, and diabetes mellitus presented with disabling angina (CCS class III-IV) despite maximally tolerated medical therapy, and after both PCI and CABG. Three years earlier, triple-vessel CAD had been diagnosed after a non-ST-elevation anterior myocardial infarction. Subsequently, the patient underwent CABG surgery with venous grafts to the left anterior descendent artery (LAD), to a marginal branch (Rm), and to the posterior descendent branch (PDA) from the right coronary artery (RCA). 

**Figure 1:**
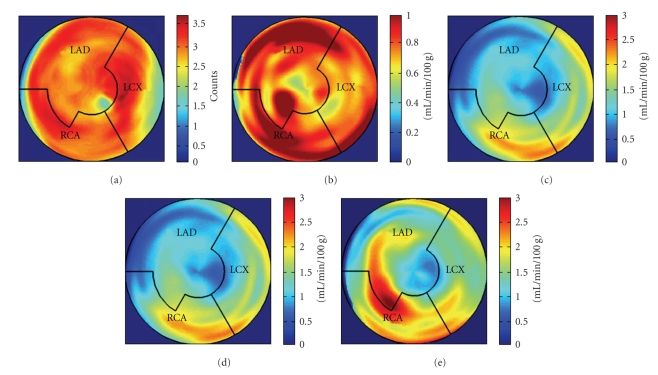
(a)–(e) PET imaging before (a)–(d) and after (e) 18 sessions of ultrasound-guided CSWT focused on the septal and inferior wall in this patient with chronic refractory angina and without a revascularization option. (a) Bulleye map of myocardial viability FDG PET at baseline, showing preserved viability (red). (b) Perfusion at rest as assessed with N-13-ammonia PET with quantitatively normal perfusion values. (c) Perfusion with N-13-ammonia under adenosine stress at baseline revealed severely reduced perfusion values in the anterior and anteroseptal wall, the apical half of the lateral wall and in the apex. (d) and (e) Blownup maps of stress perfusion at baseline (D=C) and followup (e) with improved stress perfusion in parts of the septum and the adjacent inferior wall.

During the current evaluation, echocardiography showed a preserved global left ventricular function with an ejection fraction of 55%, and without significant regional hypokinesia. A bicycle stress test was terminated at 75 watts because of dyspnoea with ST segment depression in leads V5 and V6. A repeat coronary angiography documented occlusion of the LAD, the Rm, and the RCA. The venous grafts to LAD and Rm were fully functional, the RCA bypass was occluded. There was no possibility for a repeat revascularization. Myocardial viability and perfusion at rest and stress (under adenosine induced vasodilation) were assessed by positron emission tomography (PET) imaging, with F-18-FDG and N-13-ammonia, respectively. Areas of decreased stress perfusion were found predominantly in the apex and the anterior and inferior septal wall (Figures 1(c)  and 1(d)), while viability (Figure 1(a)) and perfusion at rest (Figure 1(b)) were preserved. 

As an alternative to neurostimulator implantation the patient was offered cardiac shock wave therapy, and received 18 sessions of CSWT over a period of 6 months focused by 2D echocardiographic guidance to the most ischemic myocardial segments. Serum concentrations of cardiac enzymes (CK, CK-MB, and troponin I) were measured after each shock wave application. There was no arrhythmia during or after treatment; enzyme levels were normal at all times.

After the complete treatment course, the patient reported significant improvement of angina (CCS II), supported by an improvement in bicycle exercise capacity to 100 watt. On 2D echocardiography, no significant changes could be observed. Repeated PET imaging showed a substantial improvement of myocardial stress perfusion predominantly in the septum and the adjacent posterior wall (Figures 1(d) and 1(e)). Since the patient reported that she now was fully capable to deal with her everyday life, further treatment options were postponed.

## 3. Discussion

The medical use of electrohydraulic shock waves includes lithotripsy in urology and gastroenterology, or treatment of specific orthopaedic conditions. As compared to these applications, cardiac shock wave therapy uses shock waves emitted with a nondestructive energy level reduced by a factor of >10. In cultured endothelial cells, it was demonstrated that angiogenic factors are upregulated when exposed to these energy levels [2]. In an animal model, Nishida et al. showed that R-wave-triggered shock waves induce angiogenesis and markedly ameliorate myocardial ischemia and dysfunction without adverse effect [3]. 

In this patient, a significant improvement of angina and a moderate improvement of objective exercise tolerance could be achieved by 18 sessions of CSWT over a period of 6 months completely noninvasively, and without any adverse effect. Particularly impressive was the improvement of stress perfusion in the targeted regions as shown by PET imaging. Our case report suggests that ultrasound-guided CSWT is able to improve symptoms and perfusion in ischemic myocardium which is beyond mechanical revascularization. However, a controlled trial is needed to define the role of CSWT in the armamentarium for these challenging patients.

## References

[1] Khan SN, Dutka DP (2008). A systematic approach to refractory angina. *Current Opinion in Supportive and Palliative Care*.

[2] Gutersohn A, Gaspari G (2000). Shock waves upregulate vascular endothelial growth factor m-RNA in human umbilical vascular endothelial cells. *Circulation*.

[3] Nishida T, Shimokawa H, Oi K (2004). Extracorporeal cardiac shock wave therapy markedly ameliorates ischemia-induced myocardial dysfunction in pigs in vivo. *Circulation*.

